# Medical Journals

**Published:** 1835

**Authors:** 


### III.—ORIGINAL.

### The Western Journal.

*E Sylvis Nuncius.*

CINCINNATI APRIL 1 1835.

### I. MEDICAL JOURNALS.

In the month of January 1833, the Faculty of the Medical College of Ohio, commenced a semi-monthly Journal, under the title of the *Western Medical Gazette*, which after the 18th number was suspended for several months. Being revived, it will be continued to the completion of the second volume, when the proprietor, having taken an interest in the publication of this Journal, will discontinue that periodical, and add his subscription list to our own. His subscribers, will, therefore, be supplied with the first number of the next volume of this work, and such of them as retain it, will be considered subscribers to the Journal. By this union our subscription list will be augmented, and we shall feel under renewed obligations to Editorial exertion.

The *publishers* will hereafter be medical gentlemen; and it will be their aim and ambition to render the Journal every way commensurate with its increased patronage, and extended circulation in the Valleys of the Mississippi and the Lakes. To this end, they propose to make every practicable effort to engage the services of such medical gentlemen in the West, as by their experience in the treatment of its diseases, are qualified to communicate valuable information. By the way, why do not a greater number of our physicians record and publish the results of their practice? The habit of writing is one of the most beneficial which a medical practitioner can form; and we cannot but regret that it is not more cultivated, not merely on this account, but because of the necessity of more writers, to give a full exhibition of our medical topography, climate, peculiar diseases, and medical botany. We shall always be most happy to publish both the speculations and experience, of our Western brethren.

